# Downregulation of alpha7 nicotinic acetylcholine receptor in two-kidney one-clip hypertensive rats

**DOI:** 10.1186/1471-2261-12-38

**Published:** 2012-06-08

**Authors:** Ji-Kuai Chen, Ting Zhao, Min Ni, Dong-Jie Li, Xia Tao, Fu-Ming Shen

**Affiliations:** 1 Department of Pharmacology, School of Pharmacy, Second Military Medical University, Shanghai, 200433, China; 2 Department of Pharmacy, Changzheng Hospital, Second Military Medical University, Shanghai, 200003, China

**Keywords:** α7nAChR, 2K1C, Vagus nerve function, Tumor necrosis factor-α

## Abstract

**Background:**

Inflammation processes are important participants in the pathophysiology of hypertension and cardiovascular diseases. The role of the alpha7 nicotinic acetylcholine receptor (α7nAChR) in inflammation has recently been identified. Our previous study has demonstrated that the α7nAChR-mediated cholinergic anti-inflammatory pathway is impaired systemically in the genetic model of hypertension. In this work, we investigated the changes of α7nAChR expression in a model of secondary hypertension.

**Methods:**

The 2-kidney 1-clip (2K1C) hypertensive rat model was used. Blood pressure, vagus nerve function, serum tumor necrosis factor-α (TNF-α) and both the mRNA and protein levels of α7nAChR in tissues from heart, kidney and aorta were measured at 4, 8 and 20 weeks after surgery.

**Results:**

Compared with age-matched control, it was found that vagus nerve function was significantly decreased in 2K1C rats with the development of hypertension. Serum levels of TNF-α were greater in 2K1C rats than in age-matched control at 4, 8 and 20 weeks. α7nAChR mRNA in the heart was not altered in 2K1C rats. In the kidney of 2K1C rats, α7nAChR expression was significantly decreased at 8 and 20 weeks, but markedly increased at 4 weeks. α7nAChR mRNA was less in aorta of 2K1C rats than in age-matched control at 4, 8 and 20 weeks. These findings were confirmed at the protein levels of α7nAChR.

**Conclusions:**

Our results suggested that secondary hypertension may induce α7nAChR downregulation, and the decreased expression of α7nAChR may contribute to inflammation in 2K1C hypertension.

## Background

Secondary hypertension affects a small but significant number of the hypertensive population. It is estimated that approximately 15% of hypertensive patients have identifiable conditions causing the blood pressure elevation [1,2]. Renovascular hypertension represents the most common cause of potentially curable secondary hypertension [3]. Therefore, a better understanding of the mechanisms in the end-organ damage could provide new avenues for prevention of cardiovascular events. The 2-kidney 1-clip (2K1C) hypertensive rat is an experimental model that in many respects resembles human renovascular hypertension [4].

Inappropriately activated systemic and local tissue renin-angiotensin systems (RAS) contribute to the hemodynamic and metabolic abnormalities that lead to hypertension. Furthermore, the system can contribute to end-organ damage, at least partly by promoting inflammation [5-7]. Recent evidences indicate that neuronal cholinergic systems can modulate inflammatory responses by controlling the release of proinflammatory cytokines [8,9], and that nonneuronal acetylcholine (ACh) synthesis and release machinery are downregulated in inflammation [10]. The anti-inflammatory actions mediated by either neuronal or nonneuronal cholinergic system are believed to depend on activation of alpha7 nicotinic acetylcholine receptor (α7nAChR) [11-13], and α7nAChR is therefore proposed as an essential regulator of inflammation and a promising pharmacological strategy against infectious and inflammatory diseases [8,14]. Thus, if impairment of cholinergic pathways contribute to the development of end-organ damage during hypertension, then downregulation of the α7nAChR seems likely.

Our previous study [12] has demonstrated that α7nAChR-mediated signaling is impaired systemically in spontaneously hypertensive rats (SHR, a well-known genetic model of hypertension), which contributes to end-organ damage. Downregulation of α7nAChR occurs at the age of 20 and 40 weeks, but not 4 weeks. Therefore it seems possible that elevated blood pressure may be the fundamental cause responsible for α7nAChR downregulation regardless of the genetic factors. In the present study, using 2K1C hypertensive rats, we test the hypothesis that hypertension induces vagus nerve dysfunction and downregulation of α7nAChR in secondary hypertension, which contribute to inflammation in hypertension.

## Methods

### Animals

Male Sprague–Dawley (SD) rats (8 weeks of age; Sino-British SIPPR/BK Lad Animal Ltd, Shanghai, China) were housed in a 12/12-hour light/dark cycle with free access to food and water. All the animals used in this work received humane care in compliance with the institutional animal care guidelines and the Guide for Care and Use of Laboratory Animals published by the National Institutes of Health.

### Preparation of 2K1C

Male SD rats weighing 160 to 180 g were anesthetized with a combination of ketamine (40 mg/kg) and diazepam (6 mg/kg). The right renal artery was isolated through a flank incision, and a silver clip (0.2 mm internal gap) was placed on the renal artery, as described previously [15]. Sham-operated rats that underwent the same surgical procedure except for placement of the renal artery clip served as controls.

### Blood pressure and heart rate measurement

Systolic blood pressure (SBP), diastolic blood pressure (DBP), and heart rate (HR) were continuously recorded in conscious rats as described previously [12]. Briefly, rats were anesthetized with a combination of ketamine (40 mg/kg, *i.p.*) and diazepam (6 mg/kg, *i.p.*). A catheter was inserted into the lower abdominal aorta via the femoral artery for blood pressure (BP) measurement. Another catheter was placed into the abdominal vena cava via the femoral vein for drug administration. The catheters were filled with heparinized saline (150 IU/ml) to prevent clotting and were plugged with paraffin-filled 23-guage hypodermic needles. After a 2-day recovery period, rats were placed in a Plexiglas cage (30 cm diameter). The aortic catheter was connected to a BP transducer via a rotating swivel, which allowed the animal to move freely. Signals from the transducer were recorded by a computerized system (MPA 2000 M, Alcott Biotech Co LTD, Shanghai, China). SBP, DBP and HR were averaged beat-to-beat during the 5 min test period before and after atropine injection (0.03 mg/kg, *i.v.*), atropine induced HR changes were used to assess cardiac vagal tone.

### Determination of tumor necrosis factor-α (TNF-α) levels by ELISA

Male rats were anesthetized with a combination of ketamine (40 mg/kg, *i.p.*) and diazepam (6 mg/kg, *i.p.*). Blood samples were collected. Blood was centrifuged at 3,000 g for 15 min at 4°C to collect serum. The serum was kept at −80°C until analyzed. The levels of TNF-α were measured with commercial ELISA kits (R&D Systems, Minneapolis, MN, USA).

### RNA extraction and real-time quantitative PCR analysis

Total RNA was extracted from rat tissues using TRIzol reagent (Invitrogen) according to the instructions of the manufacturer. First-strand cDNA was amplified by PCR using specific primers for the cDNA of α7nAChR (accession no. NM_012832.3), 5' GGTCGTATGTGGCCGTTTG 3' (sense) and 5' TGCGGTTGGCGATGTAGCG 3' (antisense) and GAPDH as an internal control (accession no. NM_017008.3), 5' AGACCTCTATGCCAACACAGTGC 3' (sense) and 5' GAGCCACCAA TCCACACAGAGT 3' (antisense). Real-time quantitative PCR was performed using the Chromo4™ real-time PCR detection system (Bio-Rad) and the SYBR Premix Ex Taq Mixture (Takara) with specific primers. The PCR reactions were initiated with denaturation at 95°C for 10 s, followed by amplification with 40 cycles at 95°C for 10 s, and annealing at 60°C for 20 s (two-step method). Finally, melting curve analysis was performed from 60°C to 85°C. Data were evaluated with Opticon Monitor™ version 3.0 software. All samples were performed in triplicate. The relative expression of the target gene was normalized to the level of GAPDH in the same cDNA.

### Protein extraction and western blot analysis

Tissues were washed in ice-cold phosphate-buffered saline (PBS) and homogenized in a Tris–HCl buffer (20 mmol/L, pH 7.5) containing EDTA (2 mmol/L), NP-40 (1% w/v), Triton-100 (1% w/v), PMSF (2 mmol/L), leupeptin (50 μg/ml), aprotinin (25 μg/ml), pesptatin A (10 μg/ml) and dithiothreitol (DTT2 mmol/L). The supernatant was obtained by centrifugation at 11,000 g for 15 min at 4°C. The protein concentration was determined using a BCA Protein Assay Kit (Beyotime, China). Samples of approximately 30 μg were run on 10% SDS-PAGE. The proteins were electro-transferred to polyvinylidene difluoride (PVDF) membranes. The PVDF membranes were incubated with primary antibody (anti-α7nAChR, Sigma-Aldrich, 1:5,000) for 2 hours at 25°C and then incubated with horseradish peroxidase (HRP) conjugated secondary antibodies (1:1,000 dilution; Bethyl Laboratories, Inc., Montgomery, TX). The bound antibody was visualized on a Kodak Biomax film (Eastman Kodak, Rochester, NY) using a Supersignal substrate chemiluminescence detection kit (Pierce, Rockford, IL). The relative expression of the target protein was normalized to the level of GAPDH in the same sample [12].

### Statistical analysis

All values are expressed as means ± SEM. Results were analyzed by paired (within-group) or unpaired (between 2 groups) Student t test or ANOVA, followed by Tukey test (among 3 or more groups). Two-sided *P* < 0.05 was considered statistically significant.

## Results

### Basal arterial pressure and heart rate in 2K1C rats

Both SBP and DBP were significantly increased at 4, 8 and 20 weeks after 2K1C surgery when compared with age-matched sham-operated rats. The SBP reached a distinction about 50 mmHg at 4 and 8 weeks, and approached to 70 mmHg at 20 weeks. For DBP, the gap was less than 25 mmHg at 4 weeks and around 35 mmHg at 8 and 20 weeks. HR was not changed at 4, 8 and 20 weeks between the 2K1C and control rats (Table 1).

**Table 1:** **Blood pressure and heart rate changes induced by atropine (*i.v., 0.03 mg/kg*) between sham-operated and 2K1C rats (n = 6)**

		**Sham**				**2K1C**			
		**Pre-atropine**		**Post-atropine**		**Pre-atropine**		**Post-atropine**	
4 weeks	SBP (mmHg)	107±	2.1	108±	2.2	161±	8.5 *	163±	7.8
	DBP(mmHg)	78±	3.8	82±	3.7	102±	3.7 *	105±	3.1
	HR (beat/min)	342±	8.6	405±	6.8 ^##^	380±	16.4	422±	15.6 ^#^
8 weeks	SBP (mmHg)	105±	1.3	109±	1.6	155±	4.3 *	156±	5.5
	DBP(mmHg)	70±	1.9	73±	1.3	105±	6.2 *	107±	6.8
	HR (beat/min)	332±	4.4	395±	5.6 ^##^	336±	9.3	369±	9.3 ^#^
20 weeks	SBP (mmHg)	106±	1.1	108±	1.1	174±	4.2 *	176±	2.8
	DBP(mmHg)	72±	1.0	72±	1.3	109±	2.8 *	110±	2.0
	HR (beat/min)	335±	6.8	388±	8.9 ^##^	345±	6.2	377±	8.8 ^#^

Values are expressed as mean ± SEM. ^*^P < 0.05 *vs* aged-matched sham-operated rats (unpaired *t* test). ^#^*P* < 0.05, ^##^*P* < 0.01 *vs* Pre-atropine (paired *t* test). SBP, systolic blood pressure; DBP, diastolic blood pressure; HR, heart rate; 2K1C, two-kidney one-clip.

### Vagal tone in 2K1C rats

**Figure 1:**
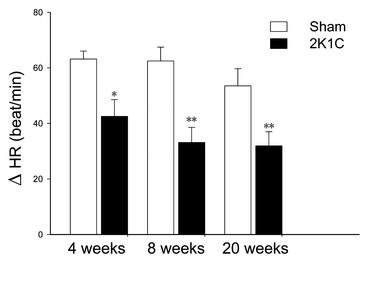
**Effects of atropine (*i.v., 0.03 mg/kg*) on heart rate in sham-operated rats and 2K1C hypertensive rats (n = 6).** ΔHR, the difference of heart rate between pre-atropine and post-atropine injection. Atropine induced HR increase in sham-operated rats was significantly larger than in 2K1C group. *P < 0.05, **P < 0.01 *vs* age-matched sham-operated group (unpaired *t* test).

To assess the vagal tone, the cardiovascular effects of atropine were compared between 2K1C and sham-operated rats. Atropine 0.03 mg/kg (*i.v.*) did not affect both SBP and DBP (Table 1). However, atropine-induced tachycardia was significantly less in 2K1C hypertensive rats than sham-operated rats at 4 weeks (42 ± 6.6 *vs.* 63 ± 2.8 beat/min; n = 6, *P* < 0.05), 8 weeks (33 ± 5.4 *vs.* 62 ± 5.0 beat/min; n = 6, *P* < 0.01), and 20 weeks (32 ± 5.1 *vs.* 54 ± 6.2 beat/min; n = 6, *P* < 0.01), indicating a deficit in cardiac vagal tone in 2K1C hypertensive rats (Figure 1).

### Inflammation induced by hypertension in 2K1C rats

**Figure 2:**
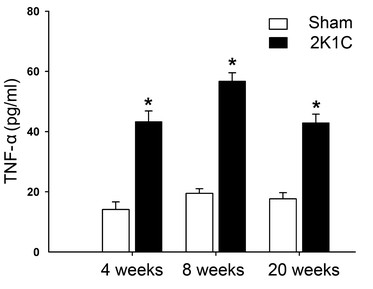
**Serum levels of TNF-α after surgery to induce 2K1C hypertension at 4, 8 and 20 weeks.** *P < 0.05 compared with aged-matched sham-operated group. Data are means ± SEM (n = 7 in each group) (unpaired *t* test).

To investigate the levels of proinflammatory cytokine in 2K1C rats, level of TNF-α in serum was measured. Serum TNF-α in 2K1C rats was significantly greater than age-matched sham-operated ones at 4 weeks (43 ± 3.5 *vs.* 14 ± 2.5 pg/ml; n = 7, *P* < 0.05), 8 weeks (57 ± 2.7 *vs.* 19 ± 1.5 pg/ml; n = 7, *P* < 0.05) and 20 weeks (43 ± 2.8 *vs.* 18 ± 2.0 pg/ml; n = 7, *P* < 0.05) (Figure 2).

### Expression of α7nAChR mRNA and its encoded protein in aorta, kidney and heart of 2K1C rats

Expression of α7nAChR mRNA and its encoded protein were determined to assess the function of cholinergic pathway in 2K1C rats.

#### Aorta

**Figure 3:**
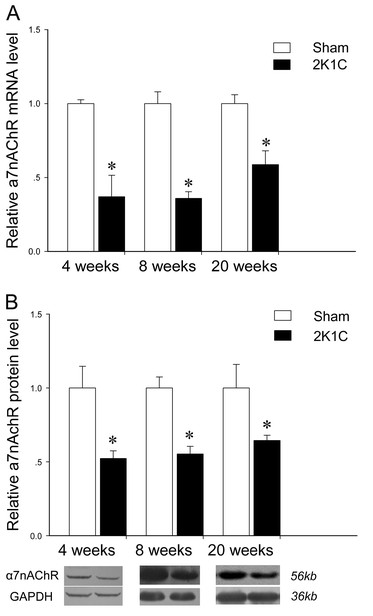
**Expression of α7nAChR on mRNA (A) and protein (B) levels in aorta of 2K1C hypertensive rats at 4 weeks, 8 weeks, and 20 weeks after surgery.** The GAPDH was used as an internal control. Data represent mean ± SEM (n = 7 in each group for real-time quantitative PCR analysis and n = 5 in each group for Western Blot analysis). *P < 0.05 *vs* aged-matched sham-operated group (unpaired *t* test).

Real-time PCR was undertaken to measure the level of mRNA. It was found that α7nAChR mRNA in aorta was significantly less in 2K1C group than in control group at 4 weeks, 8 weeks, and 20 weeks (Figure 3A). Then we investigated whether this difference was translated at the protein level. The results from Western Blot displayed that α7nAChR in aorta was markedly reduced in 2K1C hypertensive rats when compared to the sham-operated ones at 4 weeks (0.52 ± 0.05 *vs.* 1.00 ± 0.15; n = 5, *P* < 0.05), 8 weeks (0.55 ± 0.05 *vs.* 1.00 ± 0.07; n = 5, *P* < 0.05), and 20 weeks (0.64 ± 0.04 *vs.* 1.00 ± 0.16; n = 5, *P* < 0.05) (Figure 3B).

#### Kidney

**Figure 4:**
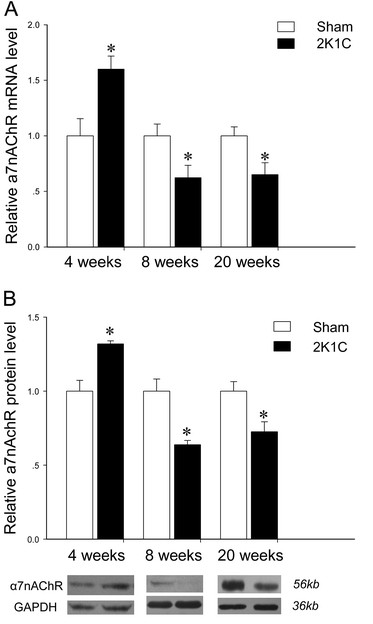
**Expression of α7nAChR on mRNA (A) and protein (B) levels in kidney of 2K1C hypertensive rats at 4 weeks, 8 weeks, and 20 weeks after surgery.** The GAPDH was used as an internal control. Data represent mean ± SEM (n = 7 in each group for real-time quantitative PCR analysis and n = 5 in each group for Western Blot analysis). *P < 0.05 *vs* aged-matched sham-operated group (unpaired *t* test).

Expression of α7nAChR mRNA in the kidney of 2K1C rats showed a temporary but significant increase at 4 weeks when compared to the control ones. However, α7nAChR mRNA in the kidney of 2K1C was less than in control group at 8 weeks and 20 weeks (Figure 4A). These results from real-time PCR were confirmed at protein levels (Figure 4B).

#### Heart

**Figure 5:**
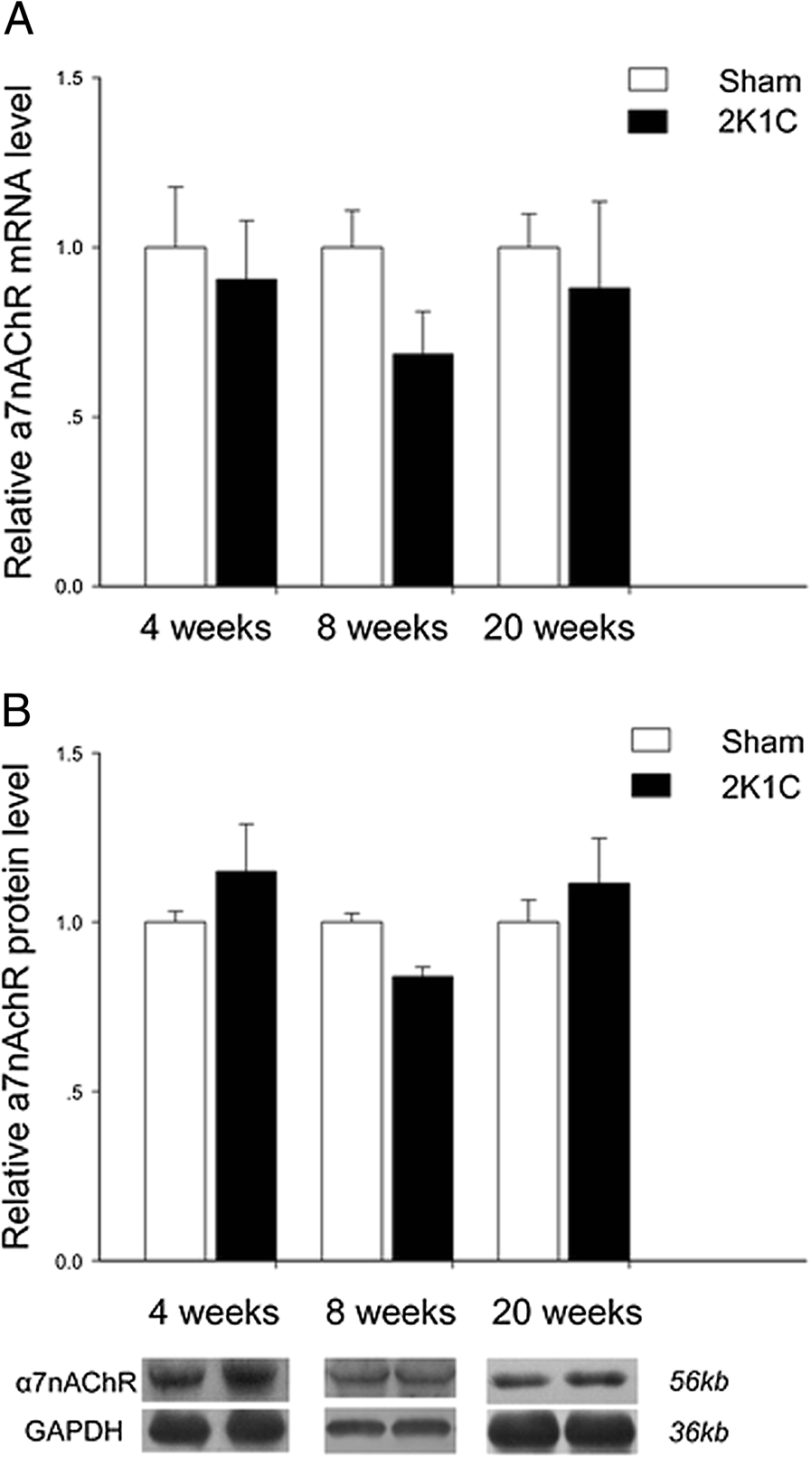
**Expression of α7nAChR on mRNA (A) and protein (B) levels in heart of 2K1C hypertensive rats at 4 weeks, 8 weeks, and 20 weeks after surgery.** The GAPDH was used as an internal control. Data represent mean ± SEM (n = 7 in each group for real-time quantitative PCR analysis and n = 5 in each group for Western Blot analysis). *P < 0.05 *vs* aged-matched sham-operated group (unpaired *t* test).

Dissimilar to results from aorta and kidney, expression of α7nAChR mRNA and its encoded protein in the tissues from the left ventricles were unchanged between the two groups at 4, 8 and 20 weeks (Figure 5).

## Discussion

In this work, we assessed the changes of cholinergic pathway with a model of secondary hypertension induced by 2K1C through determination of vagus nerve function and α7nAChR expression. We found that vagus nerve function was decreased after 2K1C surgery (*i.e.* at 4, 8 and 20 weeks of age); expression of α7nAChR was downregulated in aorta (from 4 weeks) and kidney (from 8 weeks), and serum TNF-α was increased in 2K1C hypertension.

A growing amount of evidences suggest that inflammation participates in the pathogenesis of hypertension [16,17], and hypertension may be in part an inflammatory disease because C-reactive protein level, a marker of systemic inflammation, is associated with future development of hypertension [17,18]. However, it is still unclear how hypertension is related to the inflammatory process, and what are the causes of inflammation.

It is demonstrated that hypertensive patients are characterized by a sympathovagal imbalance with a reduction of vagal tone [19,20]. Vagal function is impaired in human hypertension, which is associated with an increased risk for morbidity and mortality and may precede the development of risk factors [21]. The neuron cholinergic anti-inflammatory pathway suggests that vagus nerve can modulate the innate immune response and prevent inflammation through activation of α7nAChR in macrophages by releasing ACh, and stimulation of vagus nerve attenuates systemic inflammatory response, including inhibition of proinflammatory cytokines release, such as TNF-α and interleukin-1β [8,11,22]. Therefore, it seems reasonable that chronic hypertension results in decreased vagal function, and decreased vagal function may contribute to inflammation in hypertension. In this work, we determined the tachycardic response to atropine, a classic index of cardiac vagal tone, which reflects vagal function [23]. In accord with previous study indicating depressed cardiac vagal responsiveness in renovascular hypertensive rats [24], we found that the vagal function was significantly decreased in 2K1C hypertensive rats. These results suggested that vagus nerve might be a link between hypertension and inflammation.

It is well accepted that the α7nAChR, expressed in primary immune cells, is a pivotal mediator of the cholinergic anti-inflammatory pathway [8,9,11]. Direct activation of α7nAChR exerts a protective anti-inflammatory effects during renal ischemia/reperfusion injury [25], and regulates cytokines production in sepsis [26]. Our previous study found that chronic treatment of SHR with the α7nAChR agonist PNU-282987 relieved end-organ damage and inhibited tissue levels of pro-inflammatory cytokines [12]. Vida *et al*. suggested that cholinergic agonists inhibited systemic inflammation via the α7nAChR, and α7nAChR was a molecular link between the parasympathetic and sympathetic system to control inflammation [27]. In this study, we compared the expression of α7nAChR in the tissues from aorta, kidney and left ventricle between 2K1C hypertensive rats and control ones, and found that expression of α7nAChR was downregulated in aorta (from 4 week) and kidney (from 8 weeks). We also measured serum TNF-α, and found that levels of TNF-α in 2K1C hypertensive rats were greater than control at 4, 8 and 20 weeks. Thus decreased vagal function might play a role on the downregulation of α7nAChR, which might be responsible for the increased inflammatory cytokines in 2K1C hypertensive rats.

A main object of this study was to characterize the expression of α7nAChR in tissues from the aorta, kidney and heart in secondary hypertension induced by 2K1C. It was demonstrated that BP begins to increase 7 days after 2K1C surgery and reaches a level of 160 mmHg for SBP around 4 weeks [28-30], whereas the development of increased BP in SHR occurs largely between the ages of 4 and 12 weeks [31], and the relatively stable hypertensive levels are reached by about 16–20 weeks of age [32]. The results that α7nAChR downregulation in aorta occurred at 4 weeks in 2K1C rats and at 20 weeks in SHR suggested that BP was possibly the fundamental cause for changes of α7nAChR expression in aorta.

The RAS can contribute to end-organ damage, such as vascular remodeling, and renal fibrosis and dysfunction, at least partly by promoting inflammation [33]. It has been shown that the increased circulating Angiotensin II (ANG II) plays an important role in the development of 2K1C hypertension, and the augmentation of intrarenal ANG II levels plays the crucial role in the maintenance phase of 2K1C hypertension [34]. Hiyoshi *et al.* found that plasma renin concentrations was elevated in mice within 14 days after 2K1C surgery, followed by reduction to sham levels at 42 days. While the angiotensin II subtype 2 (AT_2_) receptor mRNA levels revealed a 2-fold increase in the thoracic aortas on day 14, and then returned to sham levels by day 42 in these mice [35]. Similar to changes of AT_2_ receptor occurred in 2K1C mice, we postulate that the upregulation of α7nAChR in the kidney at 4 weeks in our study might be a protective response to inflammation induced by Angiotensin II. Different from SHR, the expression of α7nAChR in heart of 2K1C rats was unchanged within 20 weeks, although BP remained at high levels. Further studies are needed to explore this issue.

Our results, expression of α7nAChR was downregulated in aorta (from 4 weeks) and kidney (from 8 weeks), but unchanged in heart, also suggested that the aorta may be a sensitive organ to suffer from hypertension-induced α7nAChR downregulation. Interestingly, Grundy *et al*. [18] proposed that inflammatory markers, such as high sensitive C-reactive protein in hypertension, were possibly a response to products from arterial inflammation, and hypertension at least in part was a product of arterial pathology. Though the cause of arterial inflammation is unknown, downregulated α7nAChR in the aorta during hypertension may play a role.

## Conclusion

In conclusion, downregulation of the α7nAChR occurred with the development of hypertension induced by 2K1C. Decreased expression of α7nAChR may contribute to inflammation in secondary hypertension.

## Competing interests

The authors declare that they have no competing interests.

## Authors’ contributions

Ji-Kuai Chen and Ting Zhao designed and executed the experiments, interpreted data, and wrote the manuscript. Min Ni and Dong-Jie Li performed real-time PCR and animal experiment. Fu-Ming Shen and Xia Tao conceived the study, and participated in its design and helped to draft the manuscript. All authors read and approved the final manuscript.

## Pre-publication history

The pre-publication history for this paper can be accessed here:

http://www.biomedcentral.com/1471-2261/12/38/prepub
